# Alterations in vasomotor systems and mechanics of resistance-sized mesenteric arteries from SHR and WKY male rats following *in vivo *testosterone manipulation

**DOI:** 10.1186/2042-6410-3-1

**Published:** 2012-01-03

**Authors:** Jonathan D Toot, John J Reho, Rolando J Ramirez, Jacqueline Novak, Daniel L Ely

**Affiliations:** 1 Department of Biology, 303 Carroll St.,The University of Akron, Akron, OH 44325-3908; 2 Program in Integrated Biosciences, 303 Carroll St.,The University of Akron, Akron, OH 44325-3908; 3 Department of Mathematics and Science,2020 E Maple St. Walsh University, North Canton, OH 44720-3336

**Keywords:** testosterone, norepinephrine, blood vessel, myogenic

## Abstract

**Background:**

Testosterone (T) and the sympathetic nervous system each contribute to the pathology of hypertension. Altered blood vessel reactivity is also associated with the pathology of high blood pressure. The purpose of this study was to examine the effects of T manipulation in the regulation of resistance-sized blood vessel reactivity.

**Methods:**

Adult spontaneously hypertensive (SHR) and Wistar Kyoto (WKY) male rats at 8 weeks of age were used. The rats were divided into groups consisting of gonadally intact controls (CONT), castrate with sham implant (CAST) and castrate with T implant (CAST + T) (*n *= 6 to 12 per group). Following a short-term period of T treatment (approximately 4 weeks), plasma norepinephrine (NE) and plasma T were assessed by performing high-performance liquid chromatography and RIA, respectively. Resistance-sized mesenteric artery reactivity was assessed on a pressurized arteriograph for myogenic reactivity (MYO), phenylephrine (PE) responsiveness and passive structural mechanics.

**Results:**

SHR and WKY males exhibited similar physiological trends in T manipulation, with castration significantly lowering plasma T and NE and T replacement significantly increasing plasma T and NE. T manipulation in general resulted in significant alterations in MYO of second-order mesenteric arteries, with T replacement decreasing MYO in SHR (*P *< 0.05) compared to CONT, T replacement increasing MYO, and CAST decreasing MYO in WKY rats (*P *< 0.001) compared to CONT rats. Additionally, PE-induced constriction was significantly altered in both strains following T treatment, with the effective concentration of PE to constrict the vessel to 50% of the total diameter significantly increased in the CAST + T SHR compared to CONT (*P *< 0.05). Comparisons of passive structural mechanics between SHR and WKY treatment groups indicated in SHR a significantly increased wall-to-lumen ratio and decreased circumferential wall stress compared to WKY treatment groups.

**Conclusions:**

These data suggest that T and NE are involved in a complex interaction with both myogenic reactivity and structural alterations of resistance-sized blood vessels and that these factors likely contribute to the development and maintenance of hypertension.

## Background

Studies of the etiology of hypertension have shown significant gender differences in the incidence of cardiovascular disease and related pathology [1]. These differences likely involve the sex hormones, particularly testosterone (T), which contributes to the various mechanisms involved with the development of hypertension. In addition, T (and related sex steroids) plays an integral role in hemodynamic forces, humoral interactions and the sympathetic nervous system, all of which are clearly important in cardiovascular system regulation [2]. The role of sex hormones in cardiovascular diseases is well-documented, with research widening in scope to include how various molecular and genetic signaling pathways alter vascular function [2]. Specifically, vascular function is increasingly being examined in a variety of specific *in vitro *models of isolated resistance-sized vessels, in which, following direct or indirect treatment of the vessel, T has been implicated in the pathogenesis of hypertension [3,4].

An established model of hypertension commonly studied is the spontaneously hypertensive rat (SHR), often compared to the normotensive Wistar Kyoto (WKY) rat. Strong support for potential detrimental effects of T and the protective effects of estrogen on cardiovascular function [5-7] has been demonstrated in this animal model. We have shown that prepubertal CAST lowers blood pressure in the SHR model, with T replacement restoring and even elevating blood pressure beyond gonadally intact levels [7]. These studies suggest that hypertension in SHR is a result of genetic and environmental interactions that can lead to cardiovascular pathology [2]. These data also clearly suggest an integral role of T in the regulation of blood pressure in male animals.

Reckelhoff and Granger demonstrated the role of the androgen receptor (AR) in the regulation of blood pressure [8]. Our group has shown that T and the AR in association with the SHR Y chromosome are necessary for sympathetic nervous system potentiation of blood pressure [7]. Furthermore, we have demonstrated the importance of T and the AR in the regulation of mesenteric artery reactivity in an animal model of AR deficiency (testicular feminized male) where myogenic reactivity (MYO) increased along with phenylephrine (PE) sensitivity following *in vivo *T treatment [9]. However, the role of T following *in vivo *manipulation in the regulation of resistance-sized mesenteric arteries during genetic hypertension remains unclear.

As a result, the overall objective of this study was to investigate the effects of *in vivo *T manipulation on isolated resistance-sized mesenteric artery vascular behavior. To pursue this objective, MYO, adrenergic vasoconstriction (by PE) and passive structural mechanics were examined. Therefore, the hypotheses for this investigation were that *in vivo *T manipulation will show (1) increased MYO in WKY compared to SHR males, (2) decreased adrenergic sensitivity in SHR compared to WKY males and (3) differential alteration in structural and mechanical vessel parameters in SHR and WKY males.

## Methods

### Animal model

SHR/Hsd and WKY/Hsd rats were originally obtained from Harlan Sprague-Dawley (Indianapolis, IN, USA), and colonies have been maintained at the University of Akron research facility since 1981 (SHR and WKY). All animals used in this study were from the University of Akron colony.

The overall study utilized a two-strain by three-treatment design with SHR and WKY male rats. The treatments were administered to gonadally intact controls (CONT), castrate with sham implant (CAST) and castrate with testosterone implant (CAST + T) animals, with 6 to 12 rats in each group. All protocols were reviewed and approved by the University of Akron's Institutional Animal Care and Use Committee.

All animals were pair-housed by strain and were subjected to a 12-hour:12-hour light-dark cycle and maintained on a normal sodium diet (0.3% Na, Prolab 3000 Rat Chow; PMI Feeds, St Louis, MO, USA). Food and water were accessible *ad libitum*.

### Testosterone manipulation

#### Gonadectomy

Eight-week-old male rats were sedated with 2.5% sodium pentothal (50 mg/kg intraperitoneally; Eli Lilly & Co., Indianapolis, IN, USA), and both testes were removed. In a similar manner, male rats to be T-implanted were castrated and the implant was inserted beneath the skin, between the shoulder blades and parallel to the longitudinal axis of the rat. Implants were replaced biweekly over the study duration of 4 weeks [10], with all animals being approximately 12 weeks of age when killed.

#### Testosterone implants

T implants were prepared by packing Silastic tubing (inner diameter = 1.57 mm, outer diameter = 3.17 mm) with 19 mm of testosterone propionate (Sigma-Aldrich, St Louis, MO, USA) as previously described [10]. Previous studies conducted in our laboratory [5,10,11] indicated that this level of T, although high, shows consistent results in influencing systolic blood pressure: WKY CONT (112 mmHg), WKY CAST (108 mmHg), WKY CAST + T (124 mmHg), SHR CONT (158 mmHg), SHR CAST (126 mmHg) and SHR CAST + T (185 mmHg). This produces T levels higher than normal, but we maintain that they are not out of physiological range for high normal physiological function. For instance, Toot *et al*. [10] showed that the same T implant used in this study restored sodium and potassium excretion to normal after it was increased by CAST in WKY and SHR hybrid animals. The plasma T after the implant was about the same as in the present study. CAST lowered blood pressure in both strains, and T restored the blood pressure. Plasma aldosterone followed likewise: It was increased by CAST and restored by T, suggesting that the level of T, although high, was not too high and returned electrolytes to homeostasis. Also, Sharma *et al*. [11] showed a similar blood pressure response to CAST and T replacement. They found a highly significant correlation (*r *= 0.8) between plasma T and blood pressure.

### Blood samples

Following the conclusion of the study, animals were anesthetized with 2.5% sodium pentothal prior to being killed. A 2- to 3-ml retroorbital blood sample [12] was collected between 1,100 and 1,700 hours and centrifuged for 15 minutes (5,000 × *g*) to obtain plasma for T and NE analysis. T levels were analyzed in plasma by RIA (Bio-Rad Laboratories, Hercules, CA, USA). The correlation with another kit was *r *= 0.991, sensitivity was 0.08 ng/ml at the 95% confidence limit and the highest cross-reactivity with potential interfering steroids was with 5α-dihydrotestosterone (DHT) (6.65%). The coefficient of variation for our sample intrarun was 7.4% to 11.6%, and for the interrun it was 12.5% to 16.96%.

Plasma NE was assayed by high-performance liquid chromatography with electrochemical detection (Waters 460; Waters Corp, Milford, MA, USA) [13] as previously described by investigators at our laboratory [14]. The minimum sensitivity of the NE assay was 30 pg/ml.

### Pressure arteriograph

#### Vessel isolation

After the rats were killed (overdose of 2.5% sodium pentothal), their mesenteries were placed in an ice-cold 4-(2-hydroxyethyl)-1-piperazineethanesulfonic acid (HEPES)-buffered physiological saline solution as previously described [4]. Second-order mesenteric arteries (250 to 350 μm in diameter) were isolated and mounted onto two glass cannulae in a pressurized arteriograph (Living Systems Instrumentation, Burlington, VT, USA). After residual blood was removed from the vessel, the distal cannula was occluded. The pressure arteriograph servo controller was used to maintain constant pressure of 60 mmHg for a 45-minute equilibration. The arteries were then conditioned to stretch by increasing pressure from 60 to 100 mmHg. The pressure was then returned to 60 mmHg and equilibrated for an additional 15 minutes.

#### Myogenic reactivity

By definition, MYO is the active response of an artery in response to change in intraluminal pressure. This response was investigated in an isobaric pressure arteriograph under a no-flow state. Following equilibration, the vessel was preconstricted to 70% to 85% of the initial diameter with PE (or about 25% constriction). This has previously been shown to optimize the myogenic response and induce equivalent tone in all arteries [4]. The selected intraluminal pressure was then changed in a stepwise manner from 20 to 120 mmHg in increments of 20 mmHg every 5 minutes, with only the intraluminal pressures from 60 to 120 mmHg being reported. Vessel diameters were measured with an electronic filar microscope (Lasico, Los Angeles, CA, USA). MYO was calculated as the percentage change in diameter in response to changes in intraluminal pressure [4].

MYO for each vessel was compared as the percentage change from the initial diameter at 20 mmHg as calculated using the following equation: ((*D*_x _- *D*_20_)/(*D*_20_)) × 100, where *D*_x _represents the diameter at a specific transmural pressure and *D*_20 _is the luminal diameter at 20 mmHg. A positive percentage change indicated by this equation is representative of dilation, whereas a negative percentage change is representative of constriction. Increases in MYO can be determined by a small positive or negative percentage change from the initial diameter [4]. Diameters reported in this study are for changes from 60 to 120 mmHg in the intraluminal diameter.

#### Phenylephrine constriction

A subset of resistance-sized mesenteric arteries was used to study PE constriction response. The α-adrenergic agonist PE was administered at the following cumulative concentrations of 0.1 to 7.0 μM/L. After initial diameter determination, mesenteric arteries were exposed to increasing concentrations of PE with 4 minutes between each administration to allow for a maximum response. Changes in diameter at each PE concentration were compared to the initial artery diameter as percentage constriction and then normalized as percentage maximum constriction.

#### Passive structural mechanics

A separate set of resistance-sized mesenteric arteries were analyzed under passive conditions to determine the following structural characteristics: wall thickness, wall-to-lumen ratios, wall stress and distensibility of resistance-sized mesenteric arteries. The passive HEPES buffer was made without calcium in the presence of 0.1 mM ethylene glycol tetraacetic acid and 0.1 mM papaverine to inactivate the smooth muscle of the vessel. A constant pressure of 60 mmHg was held and the vessel was equilibrated in this buffer for 30 minutes. The luminal diameter and vessel wall diameter were measured with an electronic filar and expressed in micrometers. Wall-to-lumen ratios were calculated as follows: ω/Ø_inner_, where ω is the vessel wall thickness diameter and Ø_inner _is the luminal diameter. Wall stress was calculated using the following equation (assuming the arterial wall to be uniform), assuming 1 mmHg = 1,333.2 dyn/cm^2^: T/ω, where T is equal to the intraluminal pressure multiplied by the luminal radius and ω is the vessel wall thickness. All wall stress values were multiplied by 10^5 ^to reduce scientific notation. Arterial distensibility was calculated using the following equation: ((Ø_x _- Ø_0 mmHg_) - 1) × 100. In this equation, Ø_x _is the luminal diameter at a specific intraluminal pressure and Ø_0 mmHg _is the luminal diameter at 0 mmHg and expressed as percentage distensibility [15].

### Statistics

Data were analyzed using the Sigma version 9.1 statistical software package (SigmaStat and SigmaPlot; SYSTAT Software, Inc, San Jose, CA, USA) or JMP software (Cary, NC, USA). One-way analysis of variance (ANOVA) was used to analyze each variable by treatment, as well as appropriate *t*-tests and regression analysis where applicable. Two-way ANOVA with repeated measures was used where appropriate with T treatment (CONT, CAST and CAST + T) and strain (SHR and WKY) as factors to determine the effect on plasma T and plasma NE. Statistical analysis of mesenteric artery bioassays was performed using two-way ANOVA and a *post hoc *Bonferroni test with T treatment and intraluminal pressure or PE concentration as factors. For MYO, only the intraluminal pressures of 60 to 120 mmHg were used for the statistical analyses. SigmaPlot software was used to calculate effective concentration needed to constrict to 50% of total diameter (EC_50_) values from concentrations using standard curve analysis. The EC_50 _values were analyzed using two-way ANOVA. Statistical significance was identified at a value of *P *< 0.05.

## Results

**Figure 1:**
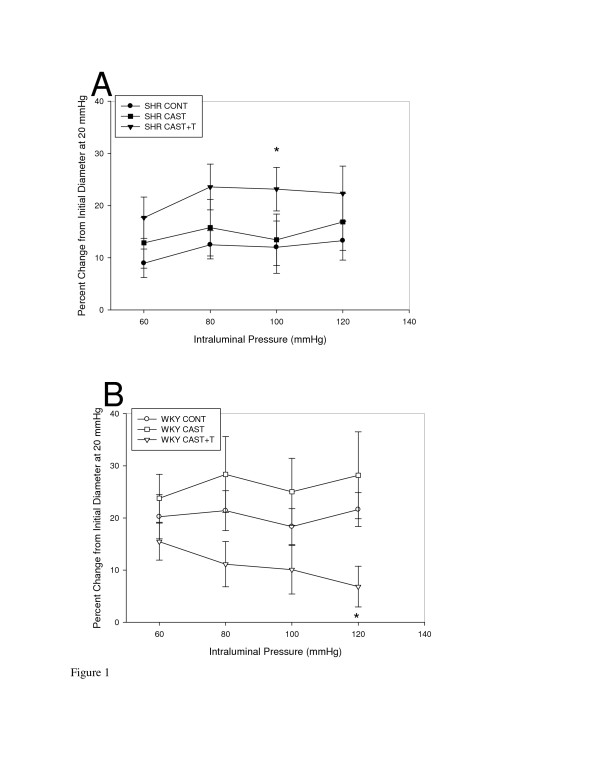
**Myogenic reactivity of resistance-sized mesenteric arteries from spontaneously hypertensive and Wistar Kyoto testosterone-manipulated male rats**. Myogenic reactivity is expressed as percentage change in diameter from an initial diameter at 20 mmHg. Only 60- to 120-mmHg pressure points are expressed in the graphs (means ± SEM). **(A) **Mesenteric arteries from spontaneously hypertensive control (SHR CONT) males displayed increased myogenic reactivity compared to SHR castrate plus testosterone (CAST + T) (*P < 0.05). **(B) **Mesenteric arteries from Wistar Kyoto (WKY) CAST + T rats displayed increased myogenic reactivity compared to WKY CONT rats (*P < 0.05).

Comparison of MYO of resistance-sized mesenteric arteries from CONT, CAST and CAST + T animals within the SHR strain (Figure 1A) demonstrated a significant treatment effect (F = 6.6, *P *< 0.05) specifically a decrease in MYO in SHR CAST + T males compared to SHR CONT males (*P *< 0.05). The comparison of WKY (Figure 1B) MYO of resistance-sized mesenteric arteries demonstrated a significant treatment effect (F = 6.2, *P *< 0.05). Specifically, the WKY CAST + T males demonstrated an increase in MYO of resistance-sized mesenteric arteries compared to WKY CONT (*P *< 0.05).

Plasma T concentrations were similar in each of the treatment groups, regardless of strain. In both SHR and WKY rats, there was a significant treatment effect (F = 5.6, *P *< 0.01) where the CAST group had the lowest plasma T and the CAST + T group had the highest plasma T (Table 1). Within strains, CAST decreased plasma T from CONT levels (*P *< 0.001) and CAST + T increased plasma T from CONT levels (*P *< 0.001) in both SHR and WKY males. In both SHR and WKY males. there was a significant NE treatment effect (F = 16.9, *P *< 0.001). The SHR CONT males had a significantly higher plasma NE compared to WKY CONT males (*P *< 0.001). No plasma NE strain differences were found between CAST and CAST + T males. In SHR, plasma NE decreased from control levels following CAST (*P *< 0.01) and T replacement increased plasma NE toward control levels (*P *< 0.05). Plasma NE followed a similar trend in WKY T-manipulated rats but did not achieve statistical significance.

**Table 1:** SHR and WKY plasma testosterone and plasma norepinephrine levels by treatment group^a^

Treatment	Strain	CONT	CAST	CAST + T
Testosterone (ng/ml)	SHR	1.88 ± 0.52	0.05 ± 0.60	12.9 ± 0.70
	WKY	1.08 ± 0.52	0.04 ± 0.70	11.0 ± 0.78
Norepinephrine (pg/ml)	SHR	970.5 ± 88.4***	481.5 ± 50.4**	762.2 ± 53.8*
	WKY	569.5 ± 28.1	415.5 ± 97.3	644.9 ± 74.6

^a^CAST, castrate; CAST + T, castrate plus testosterone; CONT, control; NE, norepinephrine; SHR: spontaneously hypertensive rat; T: testosterone; WKY: Wistar Kyoto. Data are means ± SEM. Plasma T was unaltered between SHR and WKY T-manipulated groups. Plasma NE was significantly higher in SHR CONT males than in WKY CONT males (***P < 0.001). Plasma NE was decreased in SHR CAST compared to CONT (**P < 0.01) and increased compared to CAST + T (*P < 0.05).

**Figure 2:**
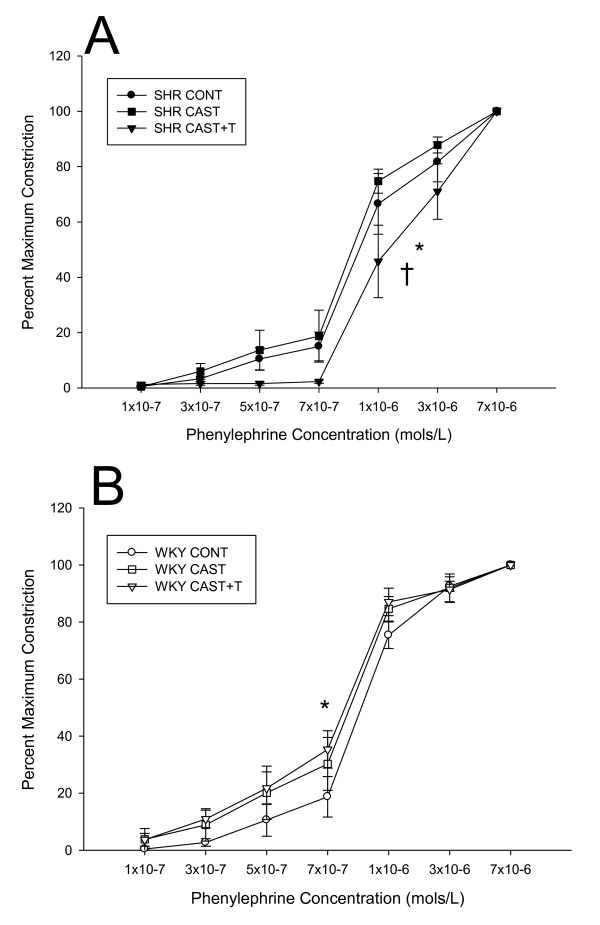
**Phenylephrine (PE) induced vasoconstriction of resistance-sized mesenteric arteries from SHR and WKY testosterone-manipulated males**. Data are expressed as percentage maximum constriction (means ± SEM). **(A) **Mesenteric arteries from spontaneously hypertensive castrate plus testosterone (SHR CAST + T) males demonstrated decreased phenylephrine (PE) constriction compared to SHR control (SHR CONT) rats (*P < 0.05) and SHR CAST rats (^†^*P *< 0.01). **(B) **Mesenteric arteries from WKY CAST + T males displayed increased PE constriction compared to WKY CONT rats (*P < 0.05).

Within the SHR strain, there was a significant treatment effect (F = 4.5, *P *< 0.05) where the PE percentage maximum constriction (Figure 2A) for SHR CAST + T males was significantly decreased compared to the SHR CONT (*P *< 0.05) and SHR CAST groups (*P *< 0.05). Within the WKY strain, there was a significant treatment effect (F = 6.9, *P *< 0.01) where PE percentage maximum constriction (Figure 2B) for WKY CAST + T rats was significantly increased compared to WKY CONT rats (*P *< 0.05). Maximal mean ± SEM contractile responses for PE were as follows: SHR CONT (60.1 ± 3.5%), SHR CAST (56.7 ± 3.9%), SHR CAST + T (43.9 ± 6.1%), WKY CONT (70.7 ± 1.7%), WKY CAST (62.0 ± 5.3%) and WKY CAST + T (66.8 ± 2.7%).

The EC_50 _levels for the second-order mesenteric arteries are presented in Table 2. There was a significant strain effect (F = 7.2, *P *< 0.05) and treatment effect (F = 3.8, *P *< 0.05). Within the SHR strain, the EC_50 _for SHR CAST + T was significantly higher compared to SHR CONT (*P *< 0.01). Within the WKY strain, WKY CONT was significantly higher compared to CAST (*P *< 0.05) and CAST + T (*P *= 0.051).

**Table 2:** Effective concentration needed to constrict to 50% of total diameter with phenylephrine in SHR and WKY treatment groups^a^

Treatment	Strain	CONT	CAST	CAST + T
EC_50_	SHR	830 ± 36 nM (*n *= 10)	827 ± 60 nM (*n *= 6)	1,570 ± 433 nM (*n *= 6)**
	WKY	844 ± 45 nM (*n *= 10)	734 ± 66 nM (*n *= 6)*	740 ± 41 nM (*n *= 6)*

^a^CAST, castrate; CAST + T, castrate plus testosterone; CONT, control; EC_50_, effective concentration needed to constrict to 50% of total diameter; SHR, spontaneously hypertensive rat; WKY: Wistar Kyoto. Data are means ± SEM. SHR CAST + T was significantly higher than SHR CONT (**P < 0.01). WKY CONT was significantly higher than CAST (*P < 0.05).

Passive structural parameters (Table 3) were determined under passive conditions (without calcium) at a constant pressure of 60 mmHg. No differences in passive inner luminal diameter were found within the SHR strain or the WKY strain. Between strains, however, the SHR CAST + T passive diameter was significantly decreased compared to WKY CAST + T (F = 7.2, *P *< 0.05). Wall thickness was unaltered within the SHR and WKY strains. Comparisons between strains, however, demonstrated a significantly thicker vessel wall between SHR CONT and WKY CONT animals (F = 5.8, *P *< 0.05). Wall-to-lumen ratios were unaltered within the SHR and WKY strains, but SHR was significantly higher than WKY in all treatment groups for wall-to-lumen ratios (F = 6.9, *P *< 0.05).

**Table 3:** Passive structural parameters of the SHR and WKY treatment groups^a^

Treatment	Strain	CONT	CAST	CAST + T
Passive diameter (μm)	SHR	305.4 ± 22.9 (*n *= 9)	285.7 ± 14.5 (*n *= 12)	++274.3 ± 6.4 (*n *= 10)
	WKY	352.8 ± 26.0 (*n *= 8)	301.9 ± 10.9 (*n *= 8)	309.6 ± 10.1 (*n *= 14)
Wall thickness (μm)	SHR	*34.9 ± 2.5 (*n *= 9)	28.4 ± 2.3 (*n *= 12)	31.7 ± 3.4 (*n *= 10)
	WKY	24.1 ± 3.8 (*n *= 8)	22.2 ± 3.5 (*n *= 8)	23.9 ± 2.7 (*n *= 10)
Wall-to-lumen ratio	SHR	*0.117 ± 0.009 (*n *= 9)	+0.102 ± 0.009 (*n *= 12)	++0.124 ± 0.014 (*n *= 10)
	WKY	0.070 ± 0.001 (*n *= 8)	0.070 ± 0.010 (*n *= 8)	0.080 ± 0.010 (*n *= 12)

**^a^**CAST, castrate; CAST + T, castrate plus testosterone; CONT, control; SHR: spontaneously hypertensive rat; T: testosterone; WKY: Wistar Kyoto. Data are means ± SEM. *P < 0.05 for SHR CONT vs WKY CONT. +^†^*P *< 0.05 for SHR CAST vs WKY CAST.++ ^‡^*P *< 0.05 for SHR CAST + T vs WKY CAST + T.

Passive mechanical parameters (Table 4) were determined under passive conditions (without calcium) at a constant pressure of 60 mmHg. Circumferential wall stress demonstrated no differences within the SHR or WKY strain. However, WKY CONT had a significantly higher wall stress than SHR CONT (F = 10.6, *P *< 0.01) and WKY CAST and CAST + T were significantly higher than SHR CAST (F = 5.1, *P *< 0.05) and CAST + T (F = 5.8, *P *< 0.05), respectively.

**Table 4:** Passive mechanical parameters from SHR and WKY treatment groups

Treatment	Strain	CONT	CAST	CAST + T
Wall stress (dyn/cm^2 ^× 10^3^)	SHR	*3.6 ± 0.3 (*n *= 9)	*4.3 ± 0.4 (*n *= 12)	*3.9 ± 0.5 (*n *= 10)
	WKY	6.9 ± 1.0 (*n *= 8)	6.2 ± 0.8 (*n *= 8)	5.9 ± 0.7 (*n *= 12)

CAST, castrate; CAST + T, castrate plus testosterone; CONT, control; NE, norepinephrine; SHR: spontaneously hypertensive rat; T: testosterone; WKY: Wistar Kyoto. Data are means ± SEM. All data were collected at 60 mmHg.*= *P *< 0.05 SHR CONT vs WKY CONT; * = *P *< 0.05 SHR CAST vs WKY CAST;*= *P *< 0.05 SHR CAST + T vs WKY CAST + T.

**Figure 3:**
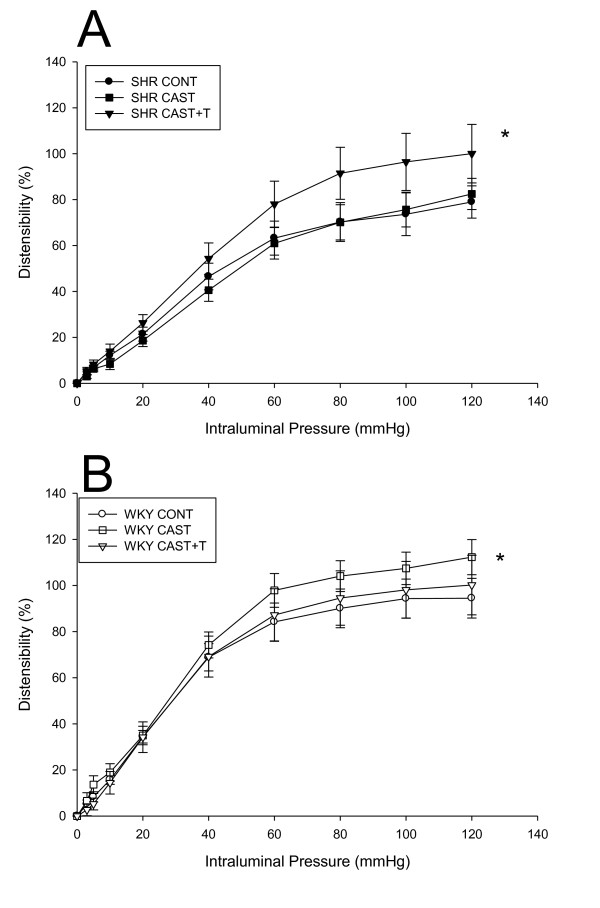
**Passive distensibility of resistance-sized mesenteric arteries from spontaneously hypertensive and Wistar Kyoto testosterone-manipulated males**. Data are expressed as percentage distensibility (means ± SEM). **(A) **Passive distensibility was increased in spontaneously hypertensive castrate plus testosterone (SHR CAST + T) mesenteric arteries compared to SHR control (SHR CONT) (* *P *< 0.01) and SHR CAST (* *P *< 0.01). **(B) **Passive distensibility was increased in Wistar Kyoto (WKY) CAST mesenteric arteries compared to WKY CONT (* *P *< 0.05) and WKY CAST + T (* *P *< 0.05).

Mesenteric artery passive distensibility was measured in the SHR and WKY T groups in calcium-free passive buffer (Figure 3). Within the SHR strain, there was a significant treatment effect (F = 8.3, *P *< 0.001), with SHR CAST + T being significantly higher than SHR CONT (*P *< 0.05) and SHR CAST (*P *< 0.05) (Figure 3A). Within the WKY strain, there was a significant treatment effect (F = 5.1, *P *< 0.05). Specifically, CAST was significantly higher than CONT (*P *< 0.05) and CAST + T (*P *< 0.05).

## Discussion

The results of this study support our hypothesis that *in vivo *manipulation of T resulted in altered regulation of MYO, PE responsiveness and passive structural mechanics from SHR and WKY males. The role of T treatment has been shown to result in nitric oxide-dependent vasodilation, vasodilation resulting from blocking the endothelial calcium channel, vasoconstriction through potassium channel activation, restoring vasoreactivity in compromised vessels or having no clear effect on vasoreactivity [16-18]. Human studies have demonstrated that acute administration of T can improve vascular reactivity and is associated with general decreases in risk factors for cardiovascular pathologies such as hypertension [19-22]. However, chronic T treatment in humans can result in increased blood pressure and risk factors for cardiovascular diseases [23-25]. Evidence reported in the literature remains inconclusive regarding the specific molecular and/or direct effects of T on the vasculature. The findings from this study mirror those of the previously discussed human data in that it has been demonstrated that T can act as a vasodilator, possibly through a combination of both genomic and nongenomic pathways [3,16,26-28]. Even with the relatively large body of research involving vessel mechanisms, evidence of the effects of *in vivo *or whole animal T treatment on vascular reactivity is currently lacking [9,29].

The primary sites of peripheral vascular resistance are the small arteries and arteriolar networks [30]. Isolated resistance caliber mesenteric arteries are useful in identifying *in vitro *vessel reactivity and structural mechanics. This study demonstrates that SHR and WKY males treated with T *in vivo *have altered reactivity of resistance-sized mesenteric arteries. The arteries from SHR CAST + T males exhibited a significant decrease in MYO compared to those taken from SHR CONT males (*P *< 0.05). Within the WKY strain, mesenteric arteries exhibited altered MYO following T manipulation, with those taken from WKY CAST + T animals displaying significant constriction in response to intraluminal pressure increases compared to WKY CONT and CAST. Incidentally, this increase in myogenic response in arteries from the WKY CAST + T rats was significantly different from that of arteries of SHR CAST + T animals. This might be a contributing factor to the higher systemic blood pressure reported in WKY CAST + T animals in our previous studies [5,10]. As T concentrations were increased beyond gonadally intact levels, T in combination with increased NE may have contributed to the increased MYO of resistance-sized mesenteric arteries in WKY CAST + T animals. Although NE levels were not significantly different by treatment in WKY animals, the trends were the same as in SHR, with some T overshoot in WKY. However, just because WKY plasma NE was not significant does not mean that it could not have an effect on interaction with the vessel wall through mechanisms described below involving a T-NE interaction. For instance, in resistance-sized cerebral arteries, Geary and colleagues found that CAST + T increased MYO of small middle cerebral arteries compared to CONT [29]. These data, in combination with those described in our previous reports [4,9], indicate a possible contribution of T in the regulation of MYO in resistance-sized mesenteric arteries. Additionally, the concept of structural autoregulation provided by Folkow [2] may play a mechanistic role in these data. The increased wall thickness in SHR CAST + T animals may require less MYO to decrease flow and may have an appreciable effect on blood pressure, whereas WKY CAST + T animals may require more MYO of mesenteric arteries to decrease flow and increase blood pressure.

CAST can have many diverse physiological effects, and one of them is reduced ARs in many tissues of the rat [31], which can be restored by androgens [32,33]. Because many genes have androgen-responsive elements, it is not surprising that CAST and T replacement have pronounced effects on gene pathways. One of the complicating factors in this study is the timing of the effects of T removal and restoration. CAST was performed when the rats were 8 weeks of age, and the T replacement lasted 4 weeks, so it is difficult to speculate about the specific effect on ARs. What we do know is that CAST seems to downregulate ARs and that T replacement appears to rapidly restore the ARs, so we assume that the ARs were restored in the T replacement group, but with the high dose of T, the exact effect is not known.

Separately, there is a specific influence of CAST and T restoration on adrenergic effectors. T has been reported to increase the α_1_-adrenergic receptors in tail arteries, whereas gonadectomy attenuates the total number of binding sites in SHR [34]. Phillipe *et al*. [35] showed a more than twofold increase in the α_1_-adrenergic receptors in response to both T and DHT. Jones *et al*. [36] showed that T restoration increased renal NE levels, which were increased by more than 100% in the presence of T in male SHR compared to no enhancement of renal NE release by T in WKY [34]. Kumai *et al*. [37] showed that CAST decreased the rate-limiting enzyme for the catecholamine tyrosine hydroxylase (TH). Likewise, Lara *et al*. [38] showed that CAST decreased the adrenergic receptor density, which was reversed with T, and they found that T regulates the adrenergic receptor gene in the SHR at the gene level [39].

Another effect is the interaction of T with adrenergic pathways. T has been shown to modulate NE tissue storage and release [5,36]. There may also be a T interaction with tissue NE which cannot be detected by plasma NE measurements alone. Mayerhofer *et al*. [40] showed that in the immature testis of the golden hamster, catecholamines acting through both α- and β-adrenergic receptors may be potent physiological stimulators of T production. Another potential T-NE interaction may occur through enhanced sympathetic outflow, which can increase blood pressure [14]. Sjöstrand and Swedin showed that androgens have no substantial direct effect on adrenergic innervation *per se*, but that they do affect the transmitter levels of the male organs indirectly through changes in the number, size and relative proportion of the target cells of the adrenergic nerves, that is, the smooth muscle cells [41]. Using males with defective ARs (testicular feminized males), our research group found that CAST decreased dopamine in several brain areas associated with blood pressure control [42]. Specifically, blood pressure correlated with dopamine levels in the medial amygdala, frontal cortex and basal nucleus of the stria terminalis. T and DHT influenced brain catecholamines in testicular feminized mutation animals, which supports the influence of a non-AR alternative mechanism. These data demonstrate an action of androgen on brain catecholamines and blood pressure that is independent of the classic AR. In future studies, we will examine the specific signaling mechanisms of T and DHT on both AR and non-AR effects on central nervous system catecholamines and γ-aminobutyric acid receptors.

Treatment of 40-day CAST rats with T restored not only the wet weights of the internal sex organs to normal but also their NE content and dopamine-β-hydroxylase activity [43]. Goldstein *et al*. showed that TH activity and TH mRNA in sympathetic ganglia was reduced after castration [44]. At the gene level, the AR transactivates the TH promoter in a ligand-dependent manner [45].

In summary, both T and adrenergic pathways have pronounced effects on many tissues and at many organizational levels (receptors, physiological pathways and genes), which can all have a significant influence on the resistance vasculature.

MYO is clearly a complex mechanism involving the integration of all three vascular layers (endothelium, smooth muscle and the extracellular matrix), as well as the chronic effects of T and NE. Researchers in studies who used isobaric vessel techniques have identified several vascular strain differences between SHR and WKY males. Specifically, SHR males have been shown to exhibit altered MYO at given pressures [46]. Izzard and colleagues were able to demonstrate differences in myogenic tone between SHR and WKY strains within the physiological range of pressure at 5 weeks of age. They also demonstrated, however, that myogenic tone generation is similar over the physiological pressure range in SHR and WKY rats at 20 weeks of age, suggesting that established hypertension leads to normalization of the myogenic response [46]. Research conducted at our laboratory has demonstrated an earlier prepubertal rise in T in SHR males, suggesting a possible role of T in the development of altered vascular reactivity in SHR males [7]. As previously indicated, however, the role of chronic *in vivo *T manipulation in the regulation of mesenteric artery reactivity in the SHR or WKY male is lacking.

Arterial responsiveness to vasoactive factors is also an important regulator of vascular reactivity. Human studies have shown that small gluteal arteries from hypertensive patients have increased sensitivity to vasoconstrictors such as NE and serotonin [47]. Resistance-sized mesenteric arteries preincubated with T displayed attenuated vasodilation in response to flutamide, suggesting that T can have vasoconstrictive effects independent of the AR [48]. Additionally, research in the A7r5 cell line has demonstrated that T can suppress calcium entry into cells by inhibition of L-type calcium channels [28] and can lead to vasoconstrictive effects by increasing thromboxane, angiotensin II, endothelin 1 and NE [36]. In small arteries from SHR animals, CAST + T treatment decreased PE vasoconstriction compared to CONT and CAST males. Interestingly, CAST + T treatment in WKY males increased PE-induced vasoconstriction compared to CONT males. These data suggest a possible sensitivity and/or functional change in the adrenergic receptor in SHR and WKY males following T manipulation. Indeed, the effective concentration required to constrict the blood vessel to 50% of its initial diameter was increased in SHR CAST + T males. The effect of T in WKY animals had the opposite effect, slightly increasing PE constriction and thereby potentially decreasing the EC_50 _value. When this is taken into account in conjunction with our passive structural data, the increased wall thickness in SHR CAST +T animals suggests that a relatively minor decrease in vessel diameter of the vasculature (that is, primary resistance vessels) will result in overall decreased in blood flow along with increased systolic blood pressure. This briefly outlined process is structural autoregulation as described in detail by Folkow [2].

Another vasoactive factor is plasma NE, which resulted in decreased PE-induced vasoconstriction in consomic SHR/y males [4]. A high-sodium diet alone without T modulation increases plasma NE in both WKY and SHR [49], and animals fed a high-sodium diet have decreased plasma NE in SHR males (33%) and WKY males (50%) after CAST- and T-normalized plasma NE [5]. Taken together, these findings support the hypothesis that T and NE contribute to the alteration of resistance vascular reactivity toward a more hypertensive phenotype. Part of the difficulty with separating the NE, T and PE data is the issue of compensation of the vessels in response to CAST or T replacement. With regard to PE constriction of resistance-sized mesenteric arteries, the significantly lowered PE constriction in SHR male rats compared to WKY rats could imply vascular remodeling or possibly downregulation of mesenteric artery α-adrenoreceptors. With regard to vascular remodeling, a decrease in the initial diameter (caused by increased resting tone or thicker vascular walls) could therefore result in a potential decrease in vasoconstriction for the vessel through a T and/or hemodynamic mechanism, as indicated in our previous studies [9,50]. Because these structural changes could potentially lead to significant changes in vessel pressure, a larger sample size would likely be necessary to further define this mechanism. An alternative explanation for the reduction in PE responses exhibited by the mesenteric arteries from the SHR could be the result of elevated plasma T and NE levels. As both T and NE can have vascular trophic effects [51,52], alterations in structural parameters, including an increased wall-to-lumen ratio, could occur.

Researchers at our laboratory have proposed a potential link through which the SHR Y chromosome can increase sympathetic nervous system activity and adrenal TH activity (rate-limiting enzyme of the catecholamine pathway), as well as an early rise in prepubertal T, all of which suggest a possible complex interaction to yield a hypertensive phenotype in SHR animals [7,14,50].

Both T and NE also exert trophic effects on the vascular wall [51,52], and T has been shown to alter gene expression in mesenteric arteries [53]. Work conducted at our laboratory has also supported the hypothesis that a relationship of the hydrostatic effect of elevated blood pressure and an elevated T increases the wall thickness of blood vessels. For instance, in male SHR from 5 to 15 weeks of age, the final blood pressure and T level showed a positive correlation with coronary artery media-to-lumen ratio and coronary collagen [54]. Also, castration reduced the media thickness by 64%, and T replacement restored the media thickness [55]. Additionally, in a normotensive model using WKY males, territorial stress increased blood pressure by 15 mmHg and T by 62%, together with a 16% increase in mesenteric vessel wall-to-lumen ratio [56]. Furthermore, investigators at our laboratory have reported that gender differences in heart disease may result from altered collagen deposition, because mesenteric collagen showed a twofold increase in males compared to age-matched females between 4 and 20 weeks of age [7,57]. Interestingly, treatment of left anterior descending fibroblasts from SHR males with T resulted in a significant increase in collagen deposition, further suggesting a role for T in the possible structural regulation of the vessel wall [57].

The findings of this study suggest that resistance-sized mesenteric artery wall-to-lumen ratios are increased in SHR males compared to WKY animals, a finding confirming the data reported by Mulvany *et al*. [58] and Bund [59]. Interestingly, wall stress of resistance-sized mesenteric arteries is decreased in SHR males compared to WKY rats, suggesting that thicker vessel walls decrease circumferential wall tension in SHR males, as shown by Folkow [2]. Furthermore, passive distensibility was altered in response to T in SHR males, demonstrating differences in extracellular matrix components in SHR mesenteric arteries. This suggests that the active responses and the structural vessel wall mechanics are altered in SHR males. This supports the hypothesis that the mechanism that changes in arterial responsiveness is related to structural changes in the arterial wall. For example, the SHR CAST + T males exhibited increased wall-to-lumen ratios, which could possibly result in less MYO needed in these arteries to significantly reduced blood flow and thus, as proposed by Folkow [2], could have an appreciable effect on systemic blood pressure.

## Conclusion

Overall, T replacement in SHR males decreased MYO, whereas T replacement in WKY males increased MYO of resistance-sized mesenteric arteries. Similarly to MYO, PE constriction was decreased in SHR males and increased in WKY male mesenteric arteries. Additionally, T treatment in SHR males increased wall-to-lumen ratios and decreased wall stress in mesenteric arteries.

## Abbreviations

AR: androgen receptor; CONT: control; CAST: castrate; CAST + T: castrate plus testosterone; EC: effective concentration; MYO: myogenic reactivity; NE: norepinephrine; PE: phenylephrine; RIA: radioimmunoassay; SEM: standard error of the mean; SHR: spontaneously hypertensive rat; T: testosterone; WKY: Wistar Kyoto.

## Competing interests

The authors declare that they have no competing interests.

## Authors' contributions

JT conducted the experiments and wrote the first draft of the manuscript. JR conducted the experiments and contributed to the first-draft manuscript and the statistics. RR and JN provided the technical training and editorial revisions. DE did the final editing. All authors reviewed and approved the final manuscript.
